# Mechanisms, assessment, and exercise interventions for skeletal muscle dysfunction post-chemotherapy in breast cancer: from inflammation factors to clinical practice

**DOI:** 10.3389/fonc.2025.1551561

**Published:** 2025-03-04

**Authors:** Pei Zhong, Xizhuang Li, Jiehua Li

**Affiliations:** ^1^ Guangxi Key Laboratory of Enhanced Recovery after Surgery for Gastrointestinal Cancer, The First Affiliated Hospital of Guangxi Medical University, Nanning, China; ^2^ Department of Gastrointestinal Gland Surgery, The First Affiliated Hospital of Guangxi Medical University, Nanning, China

**Keywords:** breast cancer post-chemotherapy, skeletal muscle dysfunction, inflammatory factors, sarcopenia, exercise intervention, muscle atrophy

## Abstract

Chemotherapy remains a central component of breast cancer treatment, significantly improving patient survival rates. However, its toxic side effects, along with cancer-related paraneoplastic syndromes, can lead to the loss of skeletal muscle mass and function, impairing physical abilities and increasing the risk of complications during treatment. Chemotherapeutic agents directly impact skeletal muscle cells by promoting protein degradation, inhibiting protein synthesis, and triggering systemic inflammation, all of which contribute to muscle atrophy. Additionally, these drugs can interfere with the proliferation and differentiation of stem cells, such as satellite cells, disrupting muscle regeneration and repair while inducing abnormal differentiation of intermuscular tissue, thereby worsening muscle wasting. These effects not only reduce the effectiveness of chemotherapy but also negatively affect patients’ quality of life and disease prognosis. Recent studies have emphasized the role of exercise as an effective non-pharmacological strategy for preventing muscle loss and preserving muscle mass in cancer patients. This review examines the clinical manifestations of muscle dysfunction following breast cancer chemotherapy, the potential mechanisms underlying these changes, and the evidence supporting exercise as a therapeutic approach for improving muscle function.

## 1 Introduction

Chemotherapy is a key component of systemic therapy for breast cancer, particularly in metastatic cases (1). Administered either orally or intravenously, chemotherapy drugs circulate throughout the body, targeting and eliminating cancer cells at multiple sites. These drugs operate through various mechanisms, including the inhibition of DNA synthesis, induction of direct DNA damage, disruption of nucleic acid synthesis, interference with topoisomerase functions, and suppression of mitosis (2). These mechanisms enable chemotherapy to eliminate a broad range of cancer cells, highlighting its wide-ranging anti-cancer efficacy. However, chemotherapy drugs have limited selectivity between normal and malignant cells. While cancer cells proliferate more rapidly and are therefore more susceptible to these drugs (3), normal cells with high turnover rates—such as those in hair follicles, bone marrow, and the gastrointestinal tract—are also affected, leading to significant side effects (4). Commonly used chemotherapeutic agents in breast cancer treatment, including anthracyclines, taxanes, and platinum-based drugs, can cause cardiotoxicity, nephrotoxicity, fatigue, cachexia, muscle atrophy, leukopenia, neutropenia, anorexia, and other gastrointestinal complications (5). These adverse effects may, in part, stem from chemotherapy-induced inflammation. Inflammatory mediators play a critical role in carcinogenesis, as chronic inflammation promotes cellular mutations and proliferation, creating an environment conducive to cancer progression (6). While chemotherapy aims to eliminate cancer cells, it can also inadvertently activate signaling pathways such as nuclear factor kappa B (NF-κB) and mitogen-activated protein kinase (MAPK), leading to an increase in pro-inflammatory mediators (7). This response triggers systemic inflammation and causes collateral damage to normal tissues, exacerbating chemotherapy-related side effects. In some instances, the doses of chemotherapy drugs used in clinical settings may be insufficient to fully suppress tumor cell growth, potentially facilitating cancer progression or recurrence (1).

Skeletal muscle dysfunction is a common side effect of chemotherapy in breast cancer patients, significantly reducing quality of life and potentially contributing to cancer-related fatigue (8). Studies have demonstrated an inverse correlation between lean body mass and chemotherapy toxicity (9), suggesting that muscle atrophy may reduce patients’ tolerance to treatment. If left unchecked, this condition can progress to sarcopenia, characterized by the gradual loss of skeletal muscle mass, strength, and function. Some researchers have suggested that sarcopenia is exclusively age-related rather than a consequence of factors such as cancer (10). However, this perspective is not entirely accurate, as multiple studies have reported a high prevalence of sarcopenia among cancer patients (11, 12), which is associated with poor prognosis (13, 14). While sarcopenia shares features with aging and cachexia (15), all forms of the condition elevate the risk of falls, fractures, physical disability, and mortality (16), reduce survival (17), and negatively influence prognosis (18). Among breast cancer patients, sarcopenia prevalence ranges from 13.9% to 32.5% (19, 20), posing a serious threat to survivors’ long-term well-being. Despite its significant impact, muscle health in post-chemotherapy breast cancer management has received limited attention, with most research focusing on cardiotoxicity. Consequently, there is a lack of routine monitoring and intervention regarding body composition, including skeletal muscle mass and lean body mass.

Growing clinical evidence supports the safety and efficacy of physical exercise as an adjunctive therapy for cancer patients (7). Exercise has been shown to regulate inflammatory factors such as interleukin-6 (IL-6) and tumor necrosis factor-alpha (TNF-α), reducing cancer-related side effects and helping restore muscle mass homeostasis (21). Even in patients with established sarcopenia, exercise can partially counteract muscle loss (22), improve resistance to chemotherapy-related complications, and improve overall physical fitness and quality of life.

This review aims to examine the effects of chemotherapy on muscle health in breast cancer patients, with a focus on muscle mass, strength, and functional capacity. It will introduce assessment methods and biochemical markers for the early detection of skeletal muscle decline, facilitating timely clinical intervention. Furthermore, the cellular mechanisms underlying muscle dysfunction in breast cancer patients will be explored to identify physiological pathways and potential therapeutic targets for preventing muscle atrophy. Finally, the impact of exercise interventions on muscle function will be discussed, along with strategies for designing structured and personalized exercise programs to counteract chemotherapy-induced muscle loss.

## 2 Clinical changes

Chemotherapy drugs have off-target effects, with 13.9% to 32.5% of breast cancer patients developing sarcopenia following treatment (19, 20). This condition is characterized by declines in both muscle mass and function. In clinical practice, various methods are used to assess muscle condition, depending on the specific needs of each case (**Table 1**). A thorough evaluation of these indicators provides insights into muscle volume, texture, and related factors, aiding in the diagnosis and monitoring of muscle deterioration.

**Table 1:** Comparison of common methods for muscle condition assessment.

Category		Assessment tool/Biochemical markers	Feasibility			Validity Data
			Advantages		Limitations	
Muscle mass Muscle quality	Simple assessment - Anthropometry	Mid-arm Muscle Circumference (MAMC)	widely used, portable, commonly applicable, inexpensive, and noninvasive, suitable for primary care settings	A surrogate marker for muscle mass, improving sensitivity in detecting sarcopenic obesity (23)	Less used in elderly populations, lower standardization (24), inability to distinguish between muscle and fat, indirect reflection of muscle mass, limited accuracy, significant individual variation	(25)
		Calf Circumference (CC)		The most commonly used anthropometric index (26). Screening indicator recommended by the Asian Working Group (27)		(28, 29)
	Precise assessment - Body Composition Analysis	Dual-energy X-ray Absorptiometry (DXA)	The most commonly used radiological tool for diagnosing sarcopenia (30), Broad applicability, Rapid scanning, Low radiation, used to calculate Appendicular skeletal muscle mass index (ASMI).		Inability to distinguish intramuscular fat, non-portable equipment, requires specialized personnel	(31, 32)
		Bioelectrical Impedance Analysis (BIA)	Convenient, rapid, non-invasive, operator-independent. Established reference values for elderly populations (33). Suitable for large-scale screening.		Limited by standardized conditions, influenced by hydration status (34), lower precision compared to DXA.	(35)
		Magnetic resonance imaging (MRI)	Non-radiative, quantifies fat, muscle, organ weight, and analyzes fat infiltration/muscle structure.		High cost, long scan time, equipment-dependent, primarily used in small-scale research requiring precise measurements.	(36–38)
		Ultrasound	Most common imaging tool for sarcopenia screening (26). Portable, radiation-free, and suitable for bedside and dynamic observation.		Lack of standardized protocols, and operator-dependent results.	(39, 40)
		Computed tomography (CT)	Measures muscle cross-sectional area and fat infiltration. Suitable for localized muscle mass assessment.		High radiation, expensive, equipment-dependent.	(36, 41)
		Peripheral quantitative computed tomography (pQCT)	Portable, low radiation. Quantifies muscle area/density in limbs. Distinguishes intermuscular and subcutaneous fat. Suitable for community-based studies with lower accuracy requirements (24).		Limited to limbs, insufficient contrast for individual muscle differentiation.	(42)
Motor functions	Assessment of Muscle Strength	Handgrip Strength Test	Classic method for upper limb strength assessment. Simple, stable data, high correlation.		Less precise, influenced by individual status.	(43, 44)
		Isokinetic Dynamometry	Measures dynamic muscle strength, high precision. Used for joint extension and angle-specific strength.		Expensive, complex operation, limited to specialized institutions.	(45)
		Chair Stand Test (CST)/Sit to Stand (STS) test	Simple, equipment-free. Assesses lower limb strength; common sarcopenia screening tool		Less precise, influenced by individual status.	(43, 44, 46)
	Physical Function	Gait Speed	Evaluates walking ability, high sensitivity, mediates sarcopenia’s impact on daily independence (47).		May be significantly influenced by comorbidities commonly present in the elderly, including degenerative or inflammatory musculoskeletal diseases (48).	(43, 49)
		6-Minute Walk Test	Assesses aerobic endurance and overall function (50). Used in chronic disease/elderly populations to evaluate sarcopenia and frailty risk.			(51, 52)
		Timed Up and Go (TUG) Test	Simple, rapid. Reflects balance, strength, flexibility. Widely used for sarcopenia screening in elderly populations.			(43, 53)
Indirect assessment - Biochemical marker		Potassium (K)	Skeletal muscle comprises approximately 60% of the total body potassium (TBK) pool, making TBK quantification a useful tool for estimating muscle mass (54). This method is entirely passive, safe, and applicable to immobile patients, as well as children, pregnant women, and other high-risk populations.		Influenced by intracellular potassium levels, nitrogen content, and the hydration coefficient of lean body mass (55).	(56)
		Serum Creatinine (SCr)	Under steady-state conditions, circulating creatinine concentration is proportional to muscle mass and serves as a reliable biomarker (57).		Lack of reference values for SCr and 24-hour creatinine excretion in the elderly (24).	(58)
		Deuterium-labeled creatine (D3-Cr)	The D3-Cr dilution method is relatively simple to perform, highly accurate, and exhibits strong consistency with whole-body muscle MRI measurements (59).		Requires specialized equipment/personnel, primarily used in epidemiological studies	(60, 61)
		Type III procollagen N-terminal peptide (P3NP/PIIINP)	By-product of type III collagen synthesis, preferred biomarker for muscle remodeling (62), associated with decreased muscle density (63)		Further research needed.	(64, 65)

### 2.1 Muscle mass

As presented in **Table 1** (Muscle Mass), multiple studies have reported significant reductions in pectoral muscle volume in breast cancer patients before and after chemotherapy (37, 38). However, muscle loss following chemotherapy does not necessarily occur uniformly across the body. Muscle biopsies from cancer patients have revealed that Type II muscle fibers are particularly susceptible to cancer-induced atrophy (66). A decline in the cross-sectional area of the vastus lateralis in breast cancer patients has been observed, paralleling the reduction in the cross-sectional area of Type II fibers (67). Interestingly, Sara Mijwel et al. noted a decrease in the proportion of Type I muscle fibers in breast cancer patients who were physically inactive during chemotherapy (68), suggesting that muscle loss may also be influenced by a reduction in overall physical activity during treatment (69, 70). Despite these findings, preclinical research on the specific types of muscle fiber atrophy induced by chemotherapy in breast cancer remains limited. Additionally, the loss of skeletal muscle mass is closely associated with poor clinical outcomes and reduced chemotherapy efficacy (71, 72), highlighting the need for further investigation into the mechanisms and patterns of muscle deterioration in these patients.

### 2.2 Muscle quality

Traditional assessments of muscle loss, particularly in the context of cachexia, present several limitations due to the confounding effects of inflammation and edema. Cachexia is frequently associated with systemic inflammation and tissue edema, leading to fluctuations in body weight and reductions in muscle mass (73). However, some of these reductions may stem from fluid retention rather than actual muscle degradation. To accurately evaluate muscle quality, it is crucial to incorporate advanced imaging techniques such as MRI, CT, or ultrasound (74–76).

One key factor influencing muscle quality is the accumulation of intermuscular adipose tissue (IMAT), a mesenchymal tissue found between muscle fibers, which has been strongly linked to muscle dysfunction (77). IMAT consists of adipocytes interspersed between muscle fibers and fascicles (78), and excessive deposition of this fat can impair muscle strength and endurance (79, 80). Notably, a preclinical study demonstrated that IMAT accumulation, even in the presence of muscle atrophy, independently compromises muscle contraction (81). Two cross-sectional studies comparing breast cancer patients with healthy controls have reported increased IMAT content in the thighs, as assessed by MRI. Furthermore, recent research has identified IMAT as a significant prognostic factor for survival outcomes in breast cancer patients, emphasizing its clinical relevance (82).

Chemotherapy also affects bone health (83). While the direct impact of chemotherapy-induced bone loss on skeletal muscle function remains unclear, substantial evidence suggests a relationship between osteoporosis and muscle mass reduction (84). This interaction may be influenced by changes in mechanical loading, as decreased muscle mass leads to reduced stress on bones, contributing to bone loss and, eventually, osteoporosis (85). However, no studies have yet examined skeletal muscle changes at different tumor progression stages in preclinical models.

### 2.3 Motor functions

**Table 1** (Motor Function) outlines various performance tests that serve as cost-effective alternatives to advanced imaging modalities for assessing skeletal muscle composition in cancer patients. Given the heterogeneity of clinical populations, establishing a universal threshold for sarcopenia remains challenging. Therefore, evaluating muscle function through performance-based tests holds substantial clinical value.

The impact of chemotherapy on muscle strength in breast cancer patients varies considerably. Some studies have reported no significant changes in the strength of large muscle groups, such as those in the upper and lower limbs (86), while others have documented notable reductions in grip strength and knee extensor strength (87). In addition to muscle strength, chemotherapy significantly affects exercise endurance in breast cancer patients (88). The 6-minute walk test (6MWT) is frequently used to assess endurance capacity (50), whereas maximal oxygen uptake (VO_2_ max), the gold standard for measuring exercise capacity, also shows a declining trend following chemotherapy (89). These findings indicate that chemotherapy may impair physical performance and endurance.

Adequate blood circulation is essential for skeletal muscle function, as it delivers oxygen and nutrients, removes metabolic waste, regulates temperature, and facilitates muscle repair and growth. Cardiopulmonary exercise testing is a valuable method for evaluating both cardiovascular endurance and muscle blood flow. Due to the cardiotoxic effects of chemotherapy drugs (90), chemotherapy-induced cardiopulmonary dysfunction appears to exacerbate cancer-related fatigue, particularly physical fatigue. This dysfunction may contribute to reduced exercise tolerance (91), further impairing overall motor function. While proper blood circulation is generally associated with stronger muscle performance, some studies have suggested that blood flow restriction (BFR) training can temporarily improve muscle strength and endurance (92, 93). However, the long-term effects of BFR interventions on overall patient health remain uncertain (94), necessitating further research to determine the safety and efficacy of such training over extended periods.

### 2.4 Biochemical indices

Over the years, biochemical markers for muscle mass assessment have been developed, offering valuable tools for the early detection and management of sarcopenia. Given the complexity of muscle wasting, relying on a single biomarker is often insufficient for an accurate diagnosis. Therefore, a combination of biochemical markers and clinical evaluations is typically necessary for a comprehensive assessment.

The serum creatinine-to-cystatin C ratio (CCR) and the Sarcopenia Index (SI), both derived from SCr and cystatin C (CysC), provide simple and objective measures for evaluating muscle mass. Abdominal CT scans, which quantify the paraspinal muscle area at the L4 level, are considered one of the gold standards for muscle mass assessment (95, 96). CCR correlates closely with paraspinal muscle mass, making it a reliable marker for estimating muscle volume (96). The SI, an extension of CCR, has been identified as a valuable biomarker for sarcopenia in cancer patients and is linked to postoperative complications and long-term survival outcomes (97, 98). Both CCR and SI exhibit a strong positive correlation with the appendicular skeletal muscle index (ASMI) and have demonstrated independent predictive value for sarcopenia in advanced cancer patients, helping to identify individuals who may benefit from targeted interventions (99). These biochemical markers offer a rapid and accessible approach for diagnosing sarcopenia, particularly in cases where kidney function remains stable.

**Figure 1:**
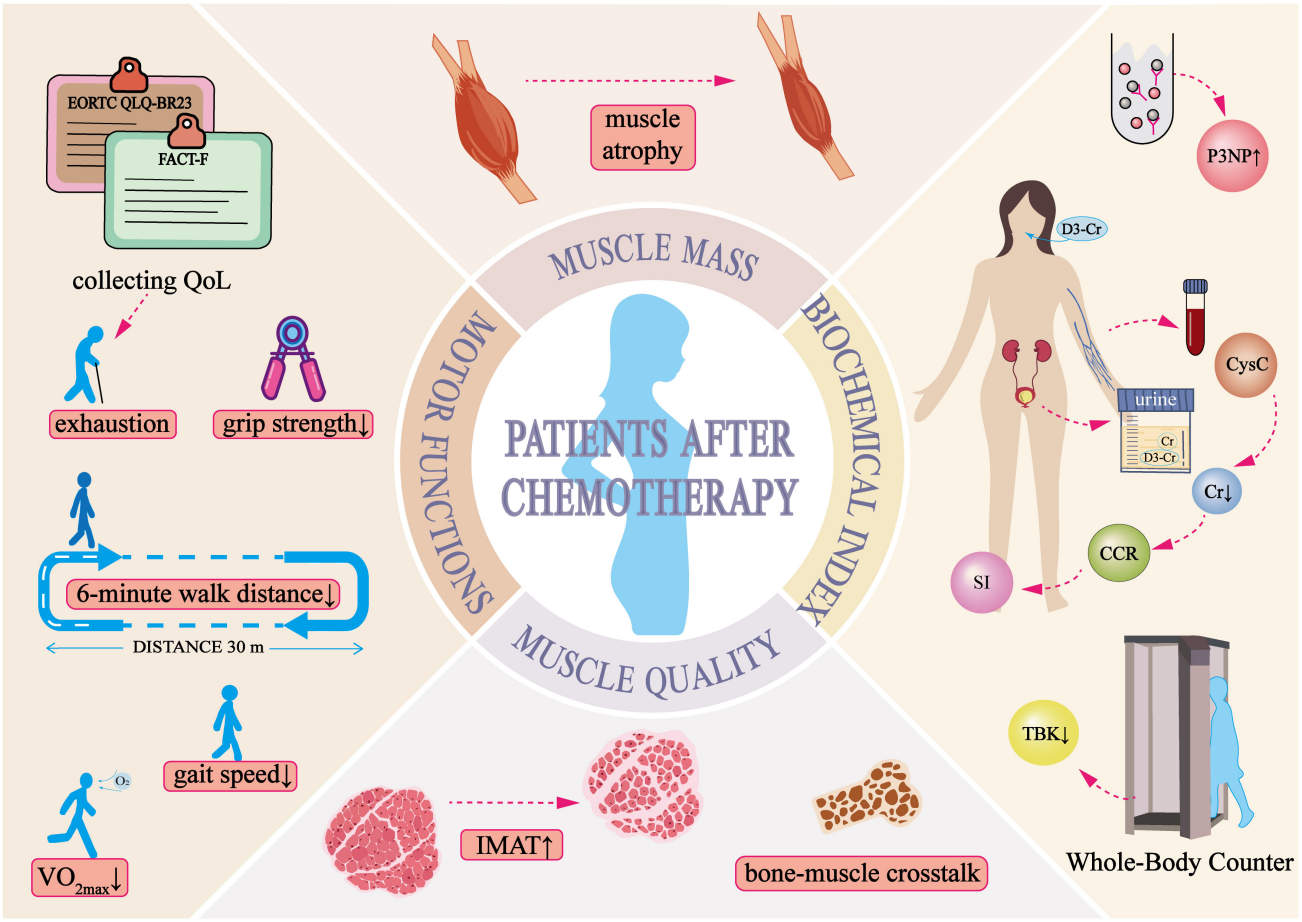
Clinical manifestations of skeletal muscle dysregulation in breast cancer patients post-chemotherapy.

The development of skeletal muscle dysfunction is closely associated with chemotherapy (45, 100). These conditions present a wide range of clinical manifestations (**Figure 1**) and significantly impact patients’ quality of life, making them a key risk factor for poor cancer prognosis (18). Despite progress in muscle mass assessment, accurately identifying muscle atrophy remains a challenge (101). Beyond the commonly observed clinical symptoms, further research is needed to develop more sensitive and specific clinical markers for detecting skeletal muscle changes and other body composition alterations during chemotherapy. Identifying high-risk patients at an early stage would allow timely intervention to slow or potentially reverse muscle loss, thereby improving both quality of life and overall prognosis.

## 3 Cellular changes

Muscle wasting in cancer patients can arise from the systemic cytotoxic effects of chemotherapy (67) or from tumor-secreted pro-inflammatory factors that disrupt skeletal muscle homeostasis by increasing proteolysis and suppressing protein synthesis (102). In the early stages of tumor development, inflammatory cells release pro-inflammatory cytokines that promote tumor growth, angiogenesis, and invasion. However, as the tumor advances, the composition of infiltrating immune cells may shift, with an increased presence of infiltrating lymphocytes (103). These lymphocytes play a crucial role in recognizing and eliminating tumor cells, and their presence is strongly associated with patient prognosis and response to immunotherapy. Despite these insights, the relationship between skeletal muscle inflammation, cancer progression, and chemotherapy remains poorly understood. A deeper understanding of these cellular alterations could help identify new therapeutic targets, potentially improving cancer treatment strategies.

### 3.1 Effects of chemotherapy drugs for breast cancer on skeletal muscle cell function

#### 3.1.1 Energy source—mitochondria

Research on muscle dysfunction in breast cancer patients has highlighted mitochondrial alterations as a key concern, particularly in response to chemotherapy. RNA sequencing (RNA-seq) studies have identified significant dysregulation of genes involved in mitochondrial function and oxidative phosphorylation in breast cancer patients (104, 105). Mitochondria, which serve as the primary energy-generating organelles, typically make up 2% to 7% of muscle cell volume (106). Chemotherapy has been shown to reduce mitochondrial numbers, as indicated by decreased citrate synthase activity (68) and direct observations from muscle biopsies, likely due to the mitotic toxicity of anticancer drugs (67). Additionally, muscle biopsies from chemotherapy-treated patients have revealed lower levels of PGC-1α protein, suggesting a decline in mitochondrial biogenesis (107).

Preclinical studies have demonstrated that doxorubicin (DOX) inhibits mitochondrial respiration (108, 109), leading to mitochondrial dysfunction. Fragmented and damaged mitochondria accumulate in skeletal muscle, reducing bioenergetic efficiency (107) and contributing to muscle weakness. Furthermore, excessive production of reactive oxygen species (ROS) (108, 110) damages healthy mitochondria (111), triggering oxidative stress at the cellular level. This oxidative stress activates protein degradation pathways, including caspase-3 and the ubiquitin-proteasome system, accelerating muscle protein breakdown (110). Additionally, increased expression of the apoptosis-related protein Bax suggests that chemotherapy-induced mitochondrial damage may lead to apoptosis, further exacerbating muscle wasting (107).

Mitochondrial network dynamics—including biogenesis, fusion, fission, and fragmentation—play a critical role in regulating muscle mass through their influence on key signaling pathways (112). Disruptions in mitochondrial fission alone are sufficient to impair organelle function and activate AMPK, ultimately leading to muscle wasting in adult animals (113). A study examining the effects of doxorubicin (DOX) on myocardial mitochondria in rats found that DOX treatment reduced the expression of fusion-related proteins (Mfn1, Mfn2, OPA1) while increasing the expression of the fission-related protein DRP1 (114). Similar findings have been reported in chemotherapy-treated breast cancer patients, where reductions in mitochondrial membrane fusion markers OPA1 (115) and Mfn2 (107) suggest a decline in mitochondrial fusion capacity. The imbalance between reduced fusion and increased fission promotes mitochondrial fragmentation (107).

Under normal physiological conditions, damaged mitochondria undergo fission and are subsequently cleared through mitophagy. However, chemotherapy appears to impair this process, as evidenced by decreased levels of PINK1 protein (115) and Parkin ubiquitin ligase (108), which may lead to the accumulation of damaged and fragmented mitochondria. This accumulation results in the excessive release of ROS and pro-apoptotic factors, further damaging muscle fibers and contributing to muscle loss (112). Additionally, mitochondrial fragmentation can activate the AMPK-FoxO3 axis, driving the expression of atrophy-related genes, accelerating protein degradation, and worsening muscle wasting (113).

#### 3.1.2 Transmission structure—myofibers

The regulation of myofibers is a dynamic process in which the balance between protein synthesis and degradation plays a crucial role. Skeletal muscle protein synthesis is primarily driven by the PI3K-Akt-mTOR pathway (116). Both clinical and preclinical studies have shown a decline in muscle protein synthesis in cancer patients, particularly in rodent models treated with DOX, where a reduction in PI3K-Akt-mTOR signaling has been observed (117). Protein degradation in skeletal muscle occurs through two major pathways: the ubiquitin-proteasome system (UPS) and the autophagy-lysosome pathway (ALP). Both pathways are regulated by FoxO transcription factors, whose excessive activation leads to significant muscle wasting (118), a key process in DOX-induced myotoxic proteolysis (119–121).

The UPS is a central protein degradation system in eukaryotic cells and plays a fundamental role in muscle atrophy (122). Increased UPS activity has been reported in cancer patients (123). In mice treated with DOX, elevated expression of FoxO1 and FoxO3 mRNA has been noted, along with increased transcription of FoxO target genes, including MAFbx/Atrogin-1 and MuRF-1 (121). The E3 ubiquitin ligases MAFbx/Atrogin-1 and MuRF-1, predominantly expressed in skeletal muscle, mediate the polyubiquitination of proteins, marking them for degradation by the 26S proteasome (124). Elevated levels of MAFbx/Atrogin-1 and MuRF-1 have been associated with muscle-wasting conditions, including cancer (125). These ligases and the subsequent proteolysis are typically upregulated when the IGF1-AKT growth-promoting pathway is inhibited (126, 127). FoxO transcription factors are key downstream targets of AKT and play a central role in regulating the expression of Atrogin-1 while driving atrophy-related pathways (126). Activation of Akt/PKB suppresses FoxO3 activity (128). In models of muscle atrophy, reduced Akt pathway activity leads to nuclear accumulation of FoxO proteins, promoting the expression of Atrogin-1/MAFbx and MuRF1, thereby accelerating protein degradation (126, 127, 129). Beyond its role in protein degradation, FoxO also influences protein synthesis. When Akt signaling is suppressed, FoxO activation downregulates mTOR, further inhibiting protein synthesis (130).

Autophagy in muscle cells contributes to increased proteolysis in cancer patients, functioning as a proteolytic mechanism activated by oxidative stress. While autophagy is essential for maintaining muscle mass by degrading damaged or aggregated proteins and facilitating baseline protein turnover (131), a more precise understanding of changes in autophagic flux is needed. Disruptions in autophagy have been linked to myofiber degeneration and atrophy, characterized by the accumulation of dysfunctional mitochondria and inclusion bodies, as seen in muscle diseases (118). The expression of autophagy-related genes (ATG), including LC3, GABARAP, and BNIP3, is regulated by FoxO3 (128). In C2C12 myotubes, FoxO3-driven proteolysis is predominantly lysosome-dependent (132). Studies have shown that DOX administration increases the expression of autophagy genes in skeletal muscle, whereas endurance exercise may provide a protective effect against DOX-induced autophagic activation (119).

Beyond these pathways, protein degradation is also influenced by fluctuations in intracellular calcium ion concentrations and the activity of inflammatory cytokines such as TNF-α. As previously noted, DOX can increase mitochondrial ROS production, which damages calcium-regulating proteins and leads to elevated intracellular calcium levels. This rise in calcium activates autophagy through calcium/calmodulin-dependent protein kinase kinase (CAMKK) and AMP-activated protein kinase (AMPK) (133, 134). In rats treated with DOX, increased oxidative stress and increased activation of proteases such as calpains and caspase-3 have been observed in skeletal muscle. However, exercise has been shown to prevent DOX-induced oxidative damage and protease activation in trained muscles (120).

Inflammatory factors also play a significant role in the proteolytic process. Activation of NF-κB has been shown to upregulate MAFbx/Atrogin-1 and MuRF1 expression in muscle atrophy models (135). Tumor-related inflammatory cytokines, such as TNF-α and IL-6, contribute to skeletal muscle atrophy by promoting protein degradation through activation of the NF-κB and UPS pathways (136, 137). In rodent models, DOX treatment has been found to elevate TNF-α levels, leading to muscle contractile dysfunction through the TNF receptor subtype TNFR1 (138).

### 3.2 Effects of chemotherapy drugs for breast cancer on skeletal muscle cell evolution

#### 3.2.1 Satellite cells, fibrosis, and abnormal deposition

The regenerative capacity of skeletal muscle is a critical factor in maintaining its function, particularly after injury (139). Successful muscle regeneration is a complex and highly coordinated process involving multiple cell types, with satellite cells (SCs) playing a central role (141). SCs are located at the myotendinous junction and beneath the basal lamina of muscle fibers (140). Studies have shown that impaired regeneration in mouse skeletal muscle is linked to insufficient SC activation and proliferation, as well as a progressive depletion of the SC pool with aging (142). In cases of muscle atrophy, muscle fibers become increasingly fragile and undergo continuous cycles of degeneration, inflammation, and impaired regeneration (143). These signs indicate persistent skeletal muscle damage, potentially leading to prolonged degeneration-regeneration cycles. The high metabolic demands of tumor proliferation can create energy shortages in non-cancerous tissues, which may alter protein turnover rates (144, 145). Unlike traditional muscle atrophy, which results primarily from protein turnover imbalances, cancer-associated muscle wasting may stem from reduced muscle repair and regeneration. Specifically, while SCs may proliferate in the cancerous environment, they often fail to differentiate properly (146), resulting in impaired muscle regeneration. Regarding the impact of chemotherapy, studies have reported that long-term DOX treatment reduces SC content and capillary density in rat skeletal muscle (147), suggesting that chemotherapy may suppress myogenic differentiation. The combined effects of cancer and chemotherapy may lead to SC dysfunction and loss in breast cancer patients, further impairing muscle regeneration. Additional research is needed to clarify how SC evolution is affected in these patients.

Fibrosis within skeletal muscle (148) and the abnormal deposition of IMAT are strong indicators of impaired muscle regeneration and pathological hallmarks of sarcopenia, contributing to muscle dysfunction (81). An increase in IMAT has been reported in breast cancer patients (115). While muscle wasting and elevated IMAT levels have been observed in chemotherapy-treated cancer models (149), research on the cellular mechanisms underlying IMAT formation and the effects of commonly used chemotherapy drugs remains limited. Fibro-adipogenic progenitor cells (FAPs) play a crucial role in skeletal muscle regeneration by maintaining tissue homeostasis and assisting SCs in responding to minor injuries (150, 151). In healthy muscle, SCs are activated at the site of damage, while FAPs proliferate to support muscle repair. These FAPs contribute to SC expansion by providing transient differentiation signals, creating an environment that enhances myogenic differentiation and facilitates muscle regeneration (152). However, under conditions of muscle disuse or disease, FAPs can differentiate into adipose or fibrotic tissue, a process widely recognized as a key contributor to IMAT accumulation (153, 154). In chemotherapy-treated breast cancer patients, studies have observed that FAPs preferentially differentiate into adipose rather than fibrotic tissue (155). In the context of skeletal muscle atrophy, research suggests that FAPs drive muscle wasting through IL-6/STAT3 signaling, and inhibiting this pathway has been shown to effectively counteract muscle atrophy and fibrosis (156). Given the dual role of FAPs in IMAT formation and muscle wasting, further studies should investigate their behavior in preclinical and clinical models of cancer-related skeletal muscle deterioration to determine their potential as therapeutic targets.

#### 3.2.2 Unfolded protein response and apoptosis

The endoplasmic reticulum (ER) is responsible for protein synthesis, folding, and modification. In the abnormal tumor microenvironment, sustained ER stress can occur in tumor cells, leading to an accumulation of misfolded or unfolded proteins (157). This accumulation activates the unfolded protein response (UPR) (158), which is thought to contribute to paraneoplastic syndromes (159). Under moderate stress conditions, the UPR acts as a protective mechanism, facilitating protein clearance through three primary pathways: the PERK-eIF2α-ATF4, IRE1α-XBP1, and ATF6-ATF6N signaling axes. These pathways upregulate genes that promote cell survival and restore cellular homeostasis. However, when ER stress is prolonged, the UPR shifts from a pro-survival response to an apoptotic signal (160).

In skeletal muscle, the UPR contributes to muscle atrophy by reducing protein synthesis, increasing protein degradation, and promoting apoptosis. Persistent UPR activation has been observed in atrophied skeletal muscle (161). This activation is regulated by E3 ubiquitin ligases such as MuRF1 and MAFbx/Atrogin-1, which facilitate protein degradation in muscle tissue (162). Among the UPR signaling pathways, PERK plays a critical role in muscle atrophy. In immobilization-induced muscle wasting models, increased expression of atrogin-1, p-PERK, and Parkin proteins, along with reduced COXIV protein levels, have been observed. Partial improvements in these markers have been reported following electrical stimulation therapy (163). Preclinical studies indicate that PERK is essential for maintaining skeletal muscle mass and function in adult mice. Deletion of PERK in tumor-bearing mice has been shown to worsen muscle atrophy, with increased activation of ubiquitin-proteasome and autophagy pathways in skeletal muscle (164).

#### 3.2.3 Myokines

Myokines are cytokines secreted by active muscles (165), exerting autocrine, paracrine, or endocrine effects (166) that contribute to muscle function and homeostasis. Various forms of exercise, particularly resistance training, have been shown to stimulate the release of myokines, which play crucial roles in anti-inflammatory, metabolic, and immune regulation (167). In cancer patients, selective atrophy of Type II muscle fibers has been observed, often accompanied by a shift from fast-twitch to slow-twitch fibers (66). DOX has also been reported to alter Type II muscle fiber composition, though endurance exercise appears to counteract these effects and provide skeletal muscle protection in mice (168). During physical activity, the secretion of myokines in response to muscle contractions is essential for preserving skeletal muscle mass (169).

Interleukin-6 (IL-6) is one of the most studied myokines, with its levels rising significantly in skeletal muscle following exercise (170). This multifunctional cytokine activates or regulates several key signaling pathways, including JAK/STAT, p38/MAPK, and NF-κB (171). IL-6 promotes SC proliferation, differentiation, and fusion (172), playing a role in muscle regeneration and contributing to skeletal muscle protein synthesis and hypertrophy. In addition to its direct effects on SCs, IL-6 promotes cell proliferation via paracrine signaling (173). However, while low IL-6 levels facilitate SC activation and muscle fiber regeneration, persistently high IL-6 levels contribute to skeletal muscle atrophy (172), which may partly explain the muscle wasting seen in cancer. As a potent pro-inflammatory cytokine, IL-6 is overexpressed in various cancers, including breast cancer (174), where abnormal activation of the IL-6/JAK/STAT3 pathway has been documented (175). Chemotherapy drugs can further activate NF-κB signaling, inducing IL-6 expression in both tumor and stromal cells (176). Studies have shown that prolonged treatment with IL-6 receptor (IL-6R) antibodies can block IL-6 signaling and reduce muscle atrophy in tumor-bearing mice, suggesting that anti-IL-6 receptor antibodies could have therapeutic potential in addressing cancer-associated muscle wasting (177). Additionally, IL-6 has been implicated in disrupting the growth hormone/insulin-like growth factor-1 (GH/IGF-1) axis, which is critical for muscle growth (178). Research has demonstrated that short-term IL-6 administration in wild-type mice (179) and humans (180) reduces circulating IGF-1 levels, thereby downregulating IGF-1/PI3K/AKT signaling, a key pathway for skeletal muscle hypertrophy.

Insulin-like growth factor 1 (IGF-1) plays a crucial role in muscle hypertrophy by activating the IGF-1/Akt/mTOR signaling pathway, which is essential for muscle growth and repair (181). However, in various forms of muscle atrophy, IGF-1 expression is downregulated, often occurring before the onset of cachexia (182). Chronic inflammation suppresses the hypothalamic GH-IGF-1 axis, leading to reduced circulating IGF-1 levels, increased protein breakdown, and the development of skeletal muscle atrophy and cachexia (183). Beyond its role in muscle maintenance, IGF-1 is vital for myogenesis, as it promotes myoblast proliferation and differentiation (184). It regulates the cell cycle of SCs and extends their replicative lifespan *in vitro*. Studies conducted *in vivo* further highlight the importance of IGF-1 signaling in SC function. Transgenic mice with skeletal muscle-specific IGF-1 overexpression exhibit increased muscle mass and strength, which correlate with enhanced SC activation and regeneration (185). In contrast, IGF-1 receptor knockout mice show impaired satellite cell function and reduced muscle regeneration (186). Exercise has been shown to activate the IGF-1/IGF-1R-PI3K/Akt signaling pathway, increasing the expression of muscle regulatory factors and promoting protein synthesis while suppressing protein degradation and apoptosis. This mechanism plays a protective role against skeletal muscle atrophy (187). In breast cancer patients undergoing chemotherapy, resistance training has been found to elevate serum IGF-1 levels, improve lean body mass, and improve muscle strength in both the upper and lower limbs. These findings suggest that targeting the IGF-1 signaling pathway may offer a promising approach for counteracting muscle atrophy and promoting muscle hypertrophy.

Myostatin, also known as growth differentiation factor 8 (GDF-8), is a member of the TGF-β family primarily expressed and secreted by skeletal muscle. It plays a critical role in regulating muscle development and adaptation in adulthood (188). As a negative regulator of muscle growth (189), myostatin inhibits protein synthesis through the myostatin-Smad2/3 signaling pathway (116). It binds to the ActRIIB receptor on the cell membrane (190), initiating the phosphorylation of Smad proteins and activating downstream intracellular signaling cascades (191, 192). By suppressing AKT phosphorylation—a key driver of skeletal muscle hypertrophy—myostatin counteracts the IGF-1/PI3K/AKT hypertrophic pathway, leading to an increase in active FoxO1 levels. This promotes the expression of atrophy-related genes such as atrogin-1, MuRF-1, and E214k (193). Additionally, Smad3 has been shown to enhance FoxO-induced expression of muscle-specific ubiquitin ligases, Atrogin-1 and MuRF-1, further accelerating muscle degradation (194). Myostatin is expressed in various tumor cell lines in both mice and humans (195), and chemotherapy has been reported to upregulate its expression, promoting catabolism and muscle atrophy (196). Research has demonstrated that systemic overexpression of myostatin in adult mice induces severe muscle and fat loss, resembling the cachexia observed in human cancer patients (197). In contrast, pharmacological inhibition of myostatin has been shown to prevent muscle loss and prolong survival (190). Clinical trials using anti-myostatin antibodies have reported increases in muscle mass and lean body mass in cancer patients with sarcopenia (198), along with improvements in functional performance (199). Additionally, alternative myostatin-targeting approaches, such as the myostatin-blocking peptide PINTA 745, have demonstrated similar benefits, increasing muscle mass and improving function in models of stroke and chronic kidney disease (200, 201).

**Figure 2:**
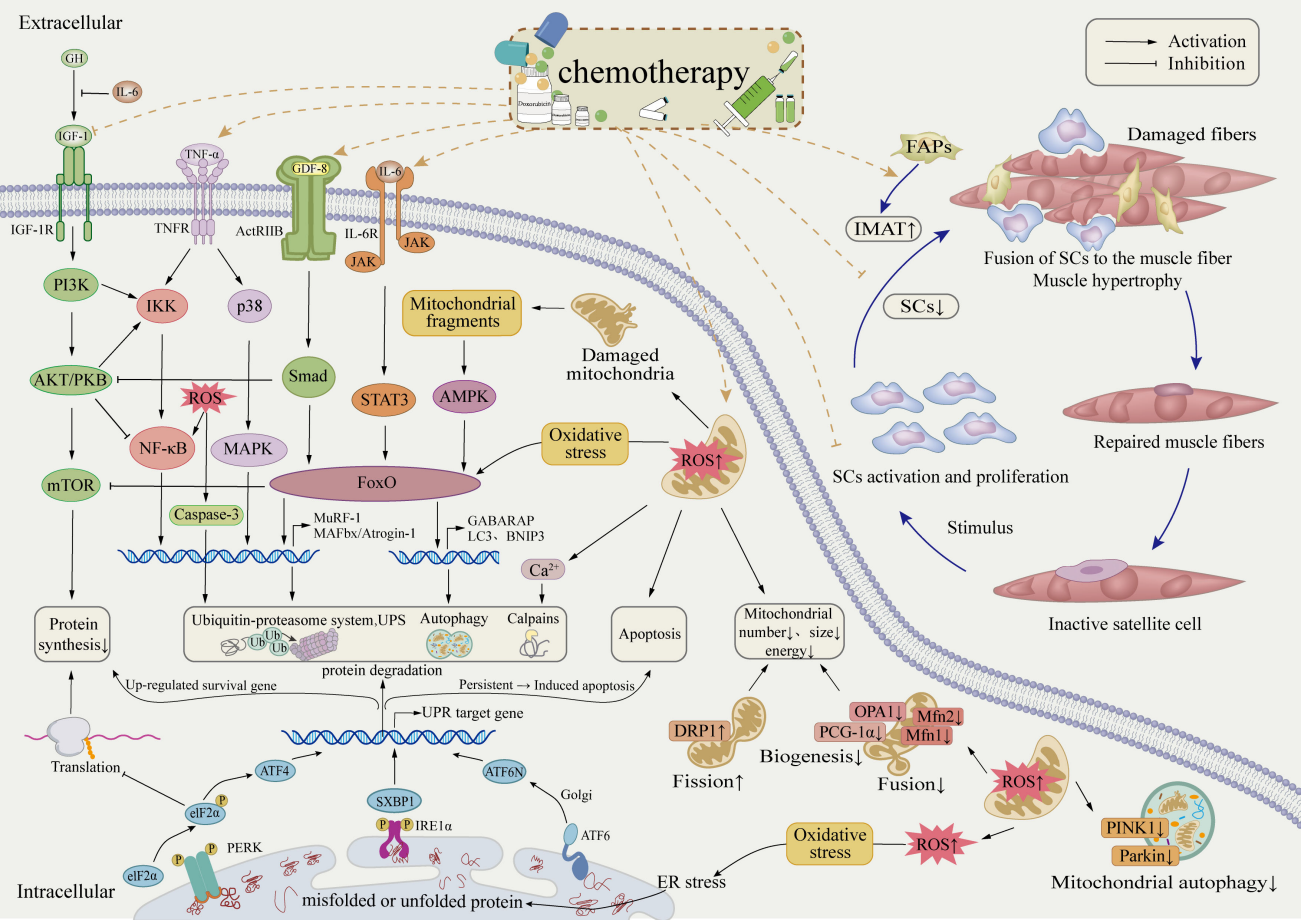
Potential cellular mechanisms of skeletal muscle dysregulation in breast cancer patients post-chemotherapy.

The cellular mechanisms underlying skeletal muscle dysfunction involve multiple interconnected pathophysiological processes (**Figure 2**). Chemotherapy disrupts mitochondrial homeostasis in skeletal muscle, leading to oxidative stress and impaired energy metabolism. It stimulates the secretion of inflammatory cytokines, triggering systemic inflammation, and interferes with key signaling pathways, including IGF-PI3K-AKT-mTOR, IL-6-JAK-STAT3, NF-κB, and MAPK. These disruptions affect the balance between muscle protein synthesis and degradation, as well as cellular autophagy. Consequently, atrophy-related genes such as atrogin-1 and MuRF-1 are upregulated, promoting protein degradation and apoptosis, ultimately compromising skeletal muscle function. In addition to its impact on protein homeostasis, chemotherapy also affects skeletal muscle cell evolution by impairing the proliferation and differentiation of endogenous stem cells. This results in abnormal muscle regeneration, defective repair, and altered intermuscular tissue differentiation, further exacerbating muscle loss. The equilibrium between protein synthesis and degradation is a fundamental mechanism in maintaining muscle mass and is regulated by complex molecular and cellular interactions. While some studies have investigated the effects of doxorubicin (DOX) on muscle, particularly cardiac muscle, further research is required to better understand skeletal muscle protein degradation in breast cancer models. This knowledge could contribute to the development of targeted interventions aimed at preserving muscle health in cancer patients.

## 4 Exercise intervention

Chemotherapy for breast cancer frequently leads to anorexia and reduced physical activity in patients (202), while also directly accelerating muscle protein degradation. Approximately 13.9%–32.5% of patients develop sarcopenia, which negatively impacts both quality of life and tolerance to treatment (19, 20). Given these consequences, effective prevention and intervention strategies are essential. Current treatments for skeletal muscle atrophy include physical exercise, nutritional supplementation, and pharmacological therapies, although no specific drug has yet been approved for clinical use (203). Preclinical studies suggest that exercise can partially counteract cisplatin-induced muscle atrophy and restore normal food intake in mice, potentially through the regulation of appetite-related hormones such as ghrelin (204). Clinical research further supports that increasing physical activity during and after breast cancer chemotherapy improves dietary intake and improves overall quality of life (205). While nutritional therapy alone may not be sufficient to significantly increase muscle mass (206, 207) and typically produces slower effects (208), its combination with exercise has been shown to enhance exercise capacity, facilitate muscle adaptation to training, and reduce muscle atrophy (209, 210).

### 4.1 Mechanisms of exercise in counteracting breast cancer-related muscle dysregulation

We reviewed research on the mechanisms by which exercise helps counteract muscle dysregulation associated with breast cancer (**Table 2**). The findings suggest that exercise plays a vital role in maintaining muscle homeostasis by improving mitochondrial function, reducing abnormal inflammatory responses, normalizing dysregulated myokine levels, and modulating the HPA axis. These processes work together to promote protein synthesis and protect skeletal muscle.

**Table 2:** Research on the mechanisms of exercise in counteracting breast cancer-related muscle dysregulation.

Reference	Study Type	Exercise Type	Intervention	Duration	Mechanism
(211)	Clinical	Aerobic exercise (AE)	Walking, cycling, etc., moderate intensity (50–70% of the age-predicted maximum heart rate) titrated to 220 min/week	12 weeks	Influences serum myokine levels
(212)	Clinical	High-intensity circuit resistance exercise (HCRE)	2-3 sessions/week, 50 minutes/session, 1 set × 8 repetitions, gradually increased from 40% to 80% of 1RM	12 weeks	Alters myokine levels, enhances NK cell activity, improves immune cell function
(213)	Clinical	Multimodal aerobic and strength training program	Supervised intermittent aerobic cycling, home walking, isometric and muscle electrical stimulation (EMS)	6 weeks	Improves muscle oxygen utilization
(214)	Preclinical	Aerobic exercise	Unsupervised running	4 weeks	↓ systemic inflammation,↑ mitochondrial biogenesis and function
(215)	Clinical	Combined aerobic and resistance training	3 sessions/week, ≈1 hour/session; resistance: 30 minutes; aerobic: starts at 10-15 minutes, increasing to 30 minutes by week 8; intensity progresses from low-moderate (weeks 1-5) to moderate-high (weeks 5-16)	16 weeks	↓ pro-inflammatory cytokines in monocytes
(216)	Clinical	HIIT and resistance training	2 sessions/week; 8 resistance exercises (2–3 sets × 8–12 repetitions at 70–80% of 1RM), followed by 3 × 3 minutes of HIIT	16 weeks	↓ systemic inflammation
(217)	Clinical	Group A: HIIT and resistance; Group B: Low-moderate aerobic and resistance	Group A: 2 sessions/week, 80–90% HRR for 2-minute intervals with 2 minutes of active rest; 3 × 6–10 RM. Group B: 2 sessions/week, HRR 40–50%, 3 × 12–20 repetitions at 50% of 6RM	6 months	↓ systemic inflammation
(218)	Clinical	HIIT	3 sessions/week, running	12 weeks	↓ low-grade inflammation, ↑ cardiopulmonary health
(219)	Clinical	Resistance training	2 sessions/week; 8 machine-based exercises; 3 sets × 8–12 repetitions at 60–80% 1RM	12 weeks	↓ systemic inflammation
(220)	Clinical	Aerobic and resistance training	3 sessions/week; aerobic at 65–80% HRmax, 30–50 minutes; resistance: 3 sets × 10 repetitions, 8 exercises at 60–80% 1RM	16 weeks	↑ mitochondrial function
(221)	Clinical	Aerobic and resistance training	3 sessions/week; 90 minutes/session; cycling and 6 resistance exercises	10 weeks	↑ protein synthesis and mitochondrial function
(68)	Clinical	HIIT and resistance training	2 sessions/week; 9 resistance exercises (2–3 sets × 8–12 repetitions at 70–80% 1RM), followed by 3 × 3 minutes of HIIT cycling at 16–18 Borg	16 weeks	↑ muscle SCs and mitochondrial function
(222)	Clinical	Aerobic exercise	Cycling at 60% VO2 peak for 30 minutes	30 minutes	Regulates HPA axis
(223)	Clinical	HIIT	3 sessions/week; 7 intervals × 30 seconds of maximum effort, with 2 minutes rest between intervals	12 weeks	Regulates HPA axis

RM, repetition maximum; HIIT, high-intensity interval training; HRR, heart rate reserve; VO_2_ peak, peak oxygen consumption; ↑, increase; ↓, decrease.

Exercise has been shown to combat systemic chronic inflammation, enhance mitochondrial function, promote satellite cell-mediated repair of damaged skeletal muscle, and stimulate protein synthesis. These effects collectively help reduce skeletal muscle atrophy in breast cancer patients after chemotherapy. Physical activity, especially resistance training, has been proven to significantly increase lean body mass (224, 225). While an increase in lean mass can counteract some of the muscle loss, cancer patients generally still have lower muscle mass compared to healthy individuals. Therefore, the goal of exercise should focus on maintaining muscle mass rather than significantly increasing it, tailored to the patient’s overall health status (226). Studies also suggest that high-intensity resistance training can enhance natural killer cell activity (NKCA) and improve immune cell function (212), implying that exercise may strengthen the immune system, which could help the patient better cope with the adverse effects of cancer and its treatments.

### 4.2 Effects of exercise on muscle improvement in breast cancer patients

In the early stages of treatment, exercise training was often discouraged due to concerns about the physical toll of chemotherapy. However, with growing evidence supporting the benefits of exercise therapy, this perspective has shifted. Increasing research suggests that exercise not only reduces treatment-related toxicity but may also enhance the effectiveness of conventional cancer therapies (227), reduce the risk of tumor recurrence and metastasis (7), and improve overall quality of life (228). As a result, exercise has become an integral component of supportive care for cancer patients, showing promise in preserving skeletal muscle mass (229, 230). Exercise programs typically include a combination of aerobic training, resistance exercises, and balance training, tailored to the patient’s specific condition and treatment stage. A review of multiple studies supports these findings (**Table 3**).

**Table 3:** Research on the muscle improvement effects of exercise in breast cancer patients.

Reference	Participants (n)	Exercise Type	Intervention	Duration	Key Outcomes
(231)	242 BC patients during chemotherapy (RE = 82, AE = 78, UC = 82)	Supervised AE and RE	AET group: cycling, treadmill, or elliptical ergometer. Weeks 1-6: 60% VO_2_ max; weeks 7-12: 70%; post-week 12: 80%. Sessions increased by 5 min every 3 weeks until reaching 45 min by week 18. RET group: 2 sets of 8-12 Reps for 9 exercises at 60-70% of estimated maximum load, increased by 10% upon completion of >12 Reps.	Chemotherapy starts 3 weeks post-chemotherapy	↑ Disease-free survival proportion, ↓ DFS event rate
(232)	40 BCS during anthracycline chemotherapy (exercise = 21, control = 19)	AE	Exercise group: moderate-to-high intensity aerobic exercise (50-90% HRmax); control group: no exercise intervention	During chemotherapy	↑ Relative VO_2_ peak, ↑ grip strength, ↑ physical activity energy expenditure, ↓ cancer-related fatigue, ↓ gastrointestinal reactions, ↓ severity of myelosuppression
(233)	93 BCS (exercise = 47, UC = 46)	AE and RE	3 sessions/week.	During chemotherapy	↓ Fatigue, ↑ appetite, ↑ HRQoL, ↑ maximum voluntary handgrip strength, ↑ lower limb strength
(234)	64 BCS during NAT	RE	Prehabilitation programs including Nordic walking, RE, and therapeutic education	From the 4th month of NAT to surgery	↓ Fatigue, ↑ arm function
(235)	Women with BC stages I-III during NAC (RT=23, UC=17)	RE	Supervised high-load strength training, 2 sessions/week	During NAC	↑ Chest and leg muscle strength
(236)	healthy women = 13, BCS = 11	RE	Progressive full-body resistance training program	12 weeks	↑ Quadriceps thickness, ↑ bench/leg/elbow press strength, ↑ grip strength
(212)	BCS(HCRE= 15、, CT= 15)	High-intensity circuit resistance exercise (HCRE)	2-3 sessions/week, 50 minutes/session, 1 set × 8 Reps, gradually increased from 40% to 80% 1RM	12 weeks	Improved BMI, body fat, muscle mass, grip strength, back muscle strength, sit-ups, whole-body reaction, single-leg stance with eyes closed, Y balance test
(237)	BCS (high-intensity = 13, moderate-intensity = 10)	AE and RE	Supervised AE, identical RE; higher intensity in the high-intensity group	16 weeks	↑ Lower limb strength, ↓ fatigue, ↓ waist circumference, ↓ neutrophil-to-lymphocyte ratio
(238)	40 BCS receiving anti-estrogen, radiotherapy, or non-hormonal systemic therapy	RE	Dose-escalated high-intensity compound exercises, 3 sessions/week	3 months	↓ Body fat percentage, ↑ muscle mass percentage, ↑ resting metabolic rate, ↑ bilateral grip strength, ↑ functional movement screen, ↑ bilateral Y balance test, ↑ Godin questionnaire scores
(239)	BCS(CT= 9, RE= 11)	RE	RE group: 3 sessions/week, 3 sets/session, 3 sets of 8-12 Reps/session at 80% of 1RM; CT group: stretching exercises, 2 sessions/week	12 weeks	↓ Fatigue, ↑ muscle strength (MP), ↑ maximum muscle strength (Pmax), ↑ lean body mass, ↑ walking speed, ↑ performance on sit-to-stand and timed-up-and-go tests
(240)	40 post-surgical BCS undergoing chemotherapy	RE combined with KT	Intervention group: KT with 2 resistance sessions/week targeting lower limbs; control: resistance training only, 2 sessions/week	12 weeks	Significant increases in hip flexor, knee extensor/flexor, ankle plantar flexor/dorsiflexor strength, SF-36 scores in both groups; greater improvements in the intervention group

BC, breast cancer; UC, usual care; RE/RET, resistance exercise/resistance exercise training; AE/AET, aerobic exercise/aerobic exercise training; CT, control group; NAT, neoadjuvant therapy; 1RM, one-repetition maximum strength; LBM, lean body mass; Pmax, maximum muscle power; MP, muscle power; MQI, muscle quality index; WS, walking speed; PPT, pain pressure threshold; BCS, breast cancer survivors; Reps, repetitions; ↑, increase; ↓, decrease; DFS, disease-free survival.

Exercise interventions have demonstrated significant benefits for breast cancer patients. Early studies suggest that engaging in physical activity can positively influence long-term disease-free survival and quality of life (QoL) outcomes (231). Compared to patients receiving standard care, those who incorporate exercise into their routine exhibit improved cardiopulmonary function, grip strength, and muscle strength in the chest, legs, and other muscle groups. Additionally, exercise has been linked to favorable changes in body composition, metabolic parameters, and muscle strength, along with higher patient-reported QoL scores. Regarding chemotherapy-related side effects, patients in exercise groups report less severe gastrointestinal disturbances and lower rates of myelosuppression compared to control groups. A recent study highlighted the added benefits of combining exercise with kinesiology taping (KT), showing greater improvements in muscle strength and QoL than exercise alone. These findings suggest that KT may serve as a non-invasive adjunct to breast cancer treatment, aiding recovery (240). Resistance training (RT) has also been investigated for its impact on pain management in breast cancer survivors. One study found that RT significantly improved one-repetition maximum strength (1RM) and pain pressure threshold (PPT) in patients experiencing persistent pain post-treatment. Notably, while strength levels remained stable after the cessation of training, PPT did not, suggesting that the analgesic effects of RT were linked to the training process itself rather than strength gains alone (241).

### 4.3 Exercise in counteracting muscle dysregulation in breast cancer patients

Exercise has been shown to improve muscle mass and function in cancer survivors, counteracting chemotherapy-induced toxicities and cancer-related muscle wasting. It may even reverse sarcopenia, significantly enhancing physical capacity, endurance, and QoL. Additionally, exercise has been associated with better chemotherapy tolerance, improved disease prognosis, and potentially extended survival (**Table 4**).

**Table 4:** Exercise interventions for muscle dysregulation in breast cancer patients.

Reference	Participants (n)	Exercise Type	Duration	Supervision	Key Outcomes
(22)	BC patients undergoing chemotherapy (RE = 66, AE = 64, UC = 70)	RE and AE	3 sessions/week, 9-24 weeks (median: 17 weeks)	Yes	RE reversed sarcopenia and deficits: ↑ SMI, ↑ muscle strength (upper and lower limb), ↑ QoL, ↑ fatigue, ↑ anemia
(242)	BCS(COMB. EX. = 50, UC = 50)	Combined AE and RE	3 sessions/week, 16 weeks	Yes	COMB. EX alleviated sarcopenic obesity phenotype: ↑ ASMI, ↓ BMI, ↓ waist circumference, ↑ lean mass, ↓ fat mass, ↓ trunk fat
(243)	Metastatic BC survivors (EX. = 49)	Gradual walking using activity tracker	Daily, 6 months	No	Improved physical performance but no changes in body composition (∼CSA, ∼Lean mass, ∼SMI); ↑ aerobic capacity, ↑ muscle strength

BC, breast cancer; BCS, breast cancer survivors; UC, usual care; RE, resistance training; AE, aerobic exercise; COMB. EX., combined exercise (aerobic and resistance); Reps, repetitions; RM, repetition maximum; HRmax, maximum heart rate; VO_2_ peak, peak oxygen consumption; ASMI, appendicular skeletal muscle mass index; SMI, skeletal muscle index; BMI, body mass index; QoL, quality of life; CSA, cross-sectional area; ↑, increase; ↓, decrease; ∼, no change.

Regular exercise has been demonstrated to enhance muscle strength and physical function in breast cancer patients undergoing chemotherapy, helping to slow or prevent further muscle loss and sarcopenia. Furthermore, even when introduced later in treatment, exercise interventions provide measurable health benefits and improve clinical outcomes. A study comparing different supervised exercise programs in obese breast cancer patients with sarcopenia undergoing adjuvant chemotherapy found that, after 17 weeks, resistance exercise (R.E.) yielded greater improvements in muscle mass and strength compared to usual care or aerobic exercise. Notably, 26.2% of participants experienced a reversal of sarcopenia, along with improvements in self-reported QoL, fatigue, and anemia (22). In addition, a combined aerobic and resistance training program performed three times per week for four months in obese breast cancer survivors with sarcopenia resulted in significant increases in the appendicular skeletal muscle mass index (ASMI). It also led to reductions in obesity-related parameters, including BMI, body weight, waist circumference, and body fat percentage, effectively improving the muscle-wasting phenotype associated with obesity (242).

Most cancer patients exhibit good tolerance to supervised exercise programs, highlighting their feasibility even in cases of sarcopenia onset. Long-term unsupervised aerobic exercise has also been shown to positively influence muscle mass, as seen in prehabilitation programs for colorectal cancer patients (244). However, supervised exercise appears to be more effective for cancer patients with sarcopenia, ensuring optimal health outcomes (245). For maximal benefit, patients should be referred to supervised exercise programs. Resistance training, in particular, has been found to be more effective in reversing sarcopenia than other exercise modalities (246–249). Among various training regimens, moderate- to high-intensity combined aerobic and resistance training has consistently shown the greatest efficacy in improving muscle mass and overall physical function (250).

Current guidelines from the American College of Sports Medicine (ACSM) (251) and the American Society of Clinical Oncology (ASCO) (252) recommend exercise both during and after cancer treatment. As previously discussed, exercise during chemotherapy provides substantial short-term benefits for patients. However, long-term follow-up studies have not demonstrated sustained improvements in fatigue reduction or the maintenance of physical function, suggesting that the benefits of exercise during treatment may decrease over time (253, 254). Despite the advantages of exercise, not all patients are suitable candidates for structured physical activity. Bedridden or severely weakened individuals may not tolerate exercise therapy, and those with advanced muscle atrophy or nerve damage may derive only minimal benefits (255, 256). Nevertheless, cancer patients are generally encouraged to increase their physical activity levels throughout and beyond treatment. Engaging in various forms of movement, even at low intensity, is preferable to complete inactivity.

## 5 Discussion

Chemotherapy remains a cornerstone of breast cancer treatment, significantly improving survival rates. However, its side effects, particularly muscle atrophy, present major challenges. Chemotherapy drugs disrupt mitochondrial homeostasis, leading to oxidative stress and impairing the balance between muscle protein synthesis and degradation. These effects are mediated through key signaling pathways, including IGF-PI3K-AKT-mTOR, IL-6-JAK-STAT3, and TNF-α-MAPK, as well as disruptions in autophagy regulation. Among commonly used chemotherapeutic agents, the myotoxicity of anthracyclines such as DOX has been extensively studied. DOX has been shown to increase ROS production in muscle fibers, induce oxidative damage, and activate proteolytic pathways, including calpains and caspase-3 (119). These mechanisms, coupled with alterations in inflammatory factors, impact satellite cells, skeletal muscle fibers, and intermuscular tissues, leading to fatigue, reduced physical function, and muscle atrophy. Despite substantial research on muscle dysfunction mechanisms, the processes underlying chemotherapy-induced muscle deterioration in breast cancer patients remain poorly understood.

The loss of skeletal muscle during chemotherapy is associated with poor prognosis in breast cancer patients (71, 72). Various assessment tools, including dynamometers, anthropometric measurements, and body composition analysis methods such as DEXA, BIA, and MRI, provide valuable diagnostic insights. However, unlike the well-defined criteria for age-related sarcopenia (16), there is no standardized diagnostic framework for chemotherapy-induced sarcopenia, particularly in breast cancer patients. Given the clinical significance of muscle loss in different contexts, a precise assessment of muscle wasting following chemotherapy is essential rather than relying on broad estimations (257). Improved diagnostic accuracy will allow for more targeted treatment strategies and better prognostic evaluations.

Exercise, as a key non-pharmacological intervention, plays an essential role in modern medical practice. It has been shown to enhance the effectiveness of chemotherapy while reducing the risk of recurrence and cancer-related mortality (7). Regarding muscle health, numerous studies suggest that exercise lowers systemic inflammation, promotes protein synthesis, and improves muscle quality and function in cancer survivors. It helps counteract chemotherapy-induced muscle atrophy and cancer cachexia and may even reverse sarcopenia. Although exercise is widely regarded as the most effective strategy for addressing skeletal muscle atrophy, it is not suitable for all patients (203). Exercise prescriptions must be tailored to individual needs, considering factors such as disease progression, treatment stage, and overall physical condition. Therefore, it is crucial to investigate how exercise modulates inflammatory responses and oxidative stress to protect muscle cells, as well as how it promotes protein synthesis and enhances mitochondrial function to improve muscle fiber strength and endurance. Integrating exercise therapy with molecular research can provide deeper insights into these mechanisms, paving the way for more personalized and scientifically based treatment strategies for breast cancer patients. In conclusion, exercise stands out as a highly effective non-pharmacological approach for addressing muscle dysfunction in breast cancer patients. Further research and development in this area are essential.
